# Perioperative outcomes of the surgical management of achalasia in two tertiary Cameroonian hospitals: a cohort study

**DOI:** 10.1186/s12876-024-03191-1

**Published:** 2024-03-22

**Authors:** Joël Igor Kamla, Guy Aristide Bang, Joel Noutakdie Tochie, George Motto Bwelle, Blondel Nana Oumarou, Bernadette Ngo Nonga

**Affiliations:** 1 Department of Surgery, University Hospital Center, Yaoundé, Cameroon; 2 Department of Surgery and Specialties, Faculty of Medicine and Biomedical Sciences, University of Yaoundé I, Yaoundé, Cameroon; 3 Anesthesia and Intensive Care Unit, Douala Laquintinie Hospital, Douala, Cameroun; 4 Visceral-Gastrointestinal Surgical unit, Yaoundé Central Hospital, Yaoundé, Cameroon; 5 Department of Visceral-Gastrointestinal and Laparoscopy surgery, National Insurance Fond Hospital, Yaoundé, Cameroon; 6 Faculty of Medicine and Biomedical Sciences, University of Yaounde I, Yaounde, Cameroon

**Keywords:** Achalasia, Heller’s cardiomyotomy, Eckardt score, Cameroon

## Abstract

**Introduction:**

Achalasia is a rare esophageal disease with potentially lethal complications. Knowledge of the outcomes of the different surgical treatment modalities for achalasia by Heller’s cardiomyotomy (HCM) helps to choose the safest and most effective option. However, data on the management of achalsia using a Heller myotomy is limited in Africa. Thus, our aim was to determine the perioperative morbidity, mortality and short-term functional outcomes of HCM in Cameroon.

**Methodology:**

We conducted a cohort study throughout a 10-year chart review of patients who underwent HCM for achalasia and were followed up postoperatively for at least three months at two tertiary health centers in Cameroon. We analyzed demographic data, preoperative clinical and imaging data, treatment details, and outcomes at three to twelve months after HCM using the Eckardt score.

**Results:**

We enrolled 29 patients with achalasia having a mean age of 24 ± 16 years and predominantly females (M/F of 1/3.8). The mean symptom duration was 51 ± 20 months. In 80% of cases, the diagnosis was made through a conventional x-ray contrast imaging or “barium swallow test” (93%) and/or an upper gastrointestinal endoscopy (86%). The gold standard diagnostic method via esophageal manometry was unavailable. Preoperatievly, all patients had symptoms suggestive of an active achalasia. HCM was performed via laparotomy in 75% as opposed to 25% laparoscopic HCM procedures. Dor’s anterior partial fundoplication was the main anti-reflux procedure performed (59%). Mucosal perforations were the only intraoperative complications in eight patients (2 during laparoscopy vs. 6 during laparotomy; *p* > 0.5) and were managed successfully by simple sutures. Postoperative complications were non-severe and occurred in 10% of patients all operated via laparotomy. The mean postoperative length of hospital stay was 7 ± 3 days for laparotomy vs. 5 ± 2 days for laparoscopy; *p* > 0.5. The perioperative mortality rate was nil. Overall, the short-term postoperative functional outcome was rated excellent; average Eckardt score of 1.5 ± 0.5 (vs. preoperative Eckardt Score of 9 ± 1; *p* < 0.0001).

**Conclusion:**

Achalasia is diagnosed late in this resource-limited setting. HCM yields satisfactory outcomes, especially via laparoscopic management. An improvement in diagnostic esophageal manometry and mini-invasive surgical infrastructure and the required surgical training/skills are needed for optimal achalasia care.

## Background

Achalasia of the cardia (AC) is a primary motor disorder of the esophagus characterized by an absence of esophageal peristaltic movements (due to a selective and irreversible decrease in esophageal submucosa innervation) as well as an absence of relaxation of the lower esophageal sphincter (LES) with consequent basal hypertonia during swallowing [1]. It occurs at incidences (per 100,000 population) of 10 North Americans [2], 0.4 to 1.1 Europeans [3], 0.27 North Africans [4] and 0.03 sub-Saharan Africans [5]. In Cameroon, some isolated cases have been reported [6, 7]. AC seems to affect both sex and to occur at any age [8]. Its diagnosis is suspected on clinical grounds of intermittent dysphagia, food regurgitation, chest pain and chronic weight loss [8]. This diagnosis is confirmed by endoscopic and radiological findings, but most importantly, using manometric studies which makes it possible to differentiate AC from other primary motor diseases of the esophagus [8, 9]. The natural history of AC is insidious over several months to years, evolving towards potentially lethal complications like aspiration pneumonitis, repeated respiratory tract sepsis, severe malnutrition and malignant transformation of the esophageal lining [10].

The treatment of AC is geared at to reducing the pressure of the LES. Heller’s cardiomyotomy (HCM) is a surgical procedure currently universally approved as the gold standard of care [11, 12]. HCM can be an open or minimally invasive surgery with no significant difference in postoperative functional outcomes [13, 14]. Most relatively large-sample size studies on AC in Africa mainly reported epidemiological, clinical and endoscopic data published seven to 40 years ago [4, 5, 15]. Very few isolated studies in the form of case reports and case series conducted in Africa report on the surgical management of AC often by HCM and mainly via laparotomy [6, 16] and lesser via laparoscopy [7]. Other treatments like peroral endoscopic myotomy, pneumatic dilatation and botulinum toxin injection at the LES are often not practiced in Africa because they are expensive and there is a scarce availability of the required technical skills and equipment. As such, HCM is often the first-line therapeutic option.

With an extensive literature search, there is a dearth of cohort data on HCM in Africa. In Cameroon, HCM has been performed via laparotomy for more than three decades [6], but laparoscopic HCM has been practiced increasingly over the last five years [7]. Knowledge of the treatment modalities of HCM and surgical outcomes of patients helps to better choose the safest and most effective therapeutic option. Therefore, we propose this study to determine the perioperative morbidity, mortality and short-term functional outcomes of HCM in two major referral hospitals in Cameroon.

## Methods

### Study design, participants and setting

We conducted a cohort study with chart review over a 10-year period spanning from July 1, 2011 to July 31, 2021 and enrolled the medical records of all patients who underwent surgery and at least three postoperative months of follow-up for AC at two Cameroonian teaching hospitals, namely the Yaoundé Central Hospital (YCH) and the National Social Insurance Fund (NSIF) Hospital. The YCH and CNPS hospital are tertiary hospitals for the referral of adult medical and surgical pathologies in Yaoundé (the political capital of Cameroon) and its environs. The study was carried out precisely at the visceral-gastrointestinal surgical units of both hospitals.

### Surgical management

**Fig. 1:**
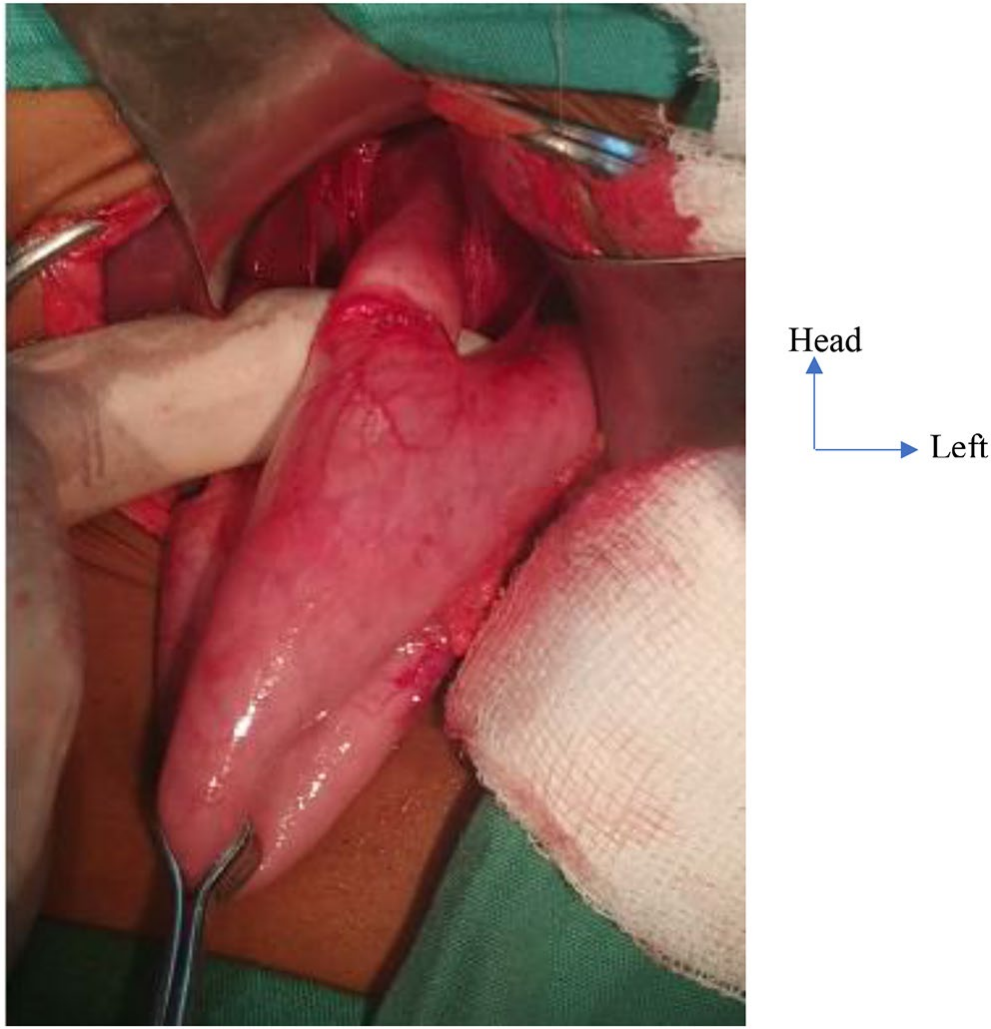
Exposure of the hiatal region in a 10-year-old girl operated by laparotomy at the Yaoundé Central Hospital, Cameroon. Circumferential stricture of the cardia with upstream dilation

**Fig. 2:**
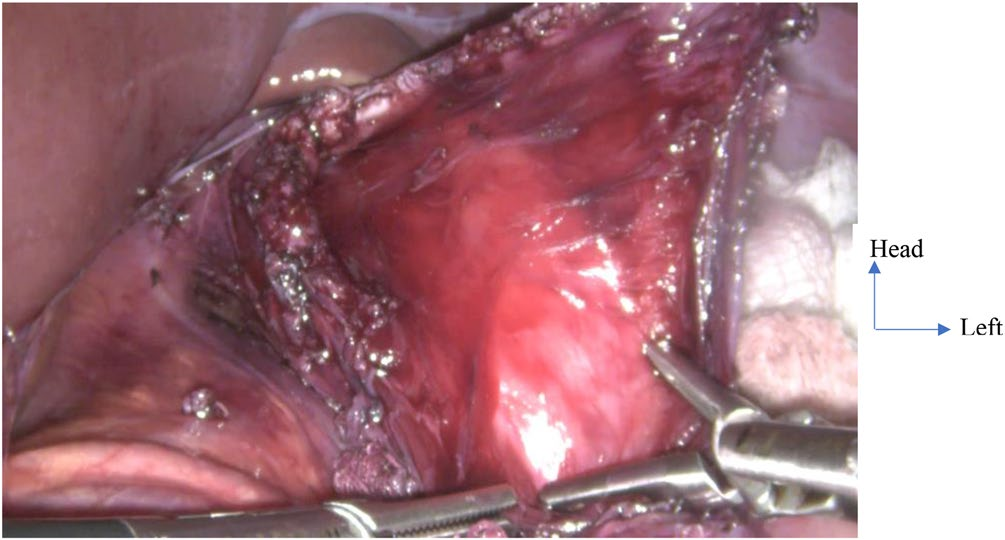
Anterior esophagocardiomyotomy in a 16-year-old girl, operated by laparoscopy in the National Insurance Fond Hospital, Yaoundé, Cameroon. The edges of the myotomy are gradually retracted to expose about one-third of the circumference of the esophagogastric mucosa with a pearly white appearance

All patients were operated on after pre-operative optimization of nutritional state and electrolyte balance by a certified anesthetist physician. A nasogastric tube was placed preoperatively to drain any stasis of esophageal fluid and/or to give a high protein diet when indicated. The surgical technique of HCM was either performed via laparotomy or laparoscopy under general anesthesia and orotracheal intubation. The surgical procedures entailed in HCM via laparotomy or laparoscopy were similar but for the surgical incisions which differed and the usual physiological changes that occurs in laparoscopy surgery. The main surgical procedures carried out during HCM via laparotomy and laparoscopy were: (i) surgical access to the hiatal region and peri-esophageal dissection; (ii) exposure of cardia of the stomach, thereby, an intraoperative assessment of tone of the LES (Fig. 1); (iii) anterior cardiomyotomy: generally, the length is 6 cm on the esophagus, 2 cm on the cardia and 2 cm on the stomach. the edges of the myotomy were surgically retracted to expose about one-third of the circumference of the esophageal mucosa; guarantee of a good myotomy (Fig. 2); (iv) making an anti-reflux procedure according to Dor, Toupet or Nissen technique; (v) the abdominal cavity was closed without drainage.

### Study variables

During the study period, we consulted the registers of operating reports, the anesthesia registers of the operating room and the hospitalization registers of the different study centers to identify patients operated on for AC. Then, we identified the files of these patients. All operated patients were contacted by phone calls for any additional relevant information after obtaining their consent. The variables analyzed were: (i) Initial symptoms (such as dysphagia, retrosternal pain, regurgitation and weight loss all assessed on Eckardt score [9], see Tables 1 and 2) and symptoms duration, defined as the time elapsed between initial symptom onset and the diagnosis of achalasia, made to define the diagnostic delay. (ii) Findings of diagnostic imaging studies namely standard chest X-ray, upper gastrointestinal endoscopy (upper GI endoscopy), barium swallow and esophageal manometry. (iii) Therapeutic details namely: past treatments, the surgical indication, the ASA grade of the patient, the surgical approach (laparotomy or laparoscopy), the total intraoperative time of the myotomy, the type of antireflux procedure and intraoperative complications. (iv) Follow-up details for at least three postoperative months; the existence or not of a repeat barium swallow and/or upper GI endoscopy, the time taken to remove the nasogastric tube, the time taken for symptoms to disappear after the start of oral enteral nutrition, early and late post-operative complications graded using the Clavien-Dindo classification (Table 3) [17], the length of hospital stay, the mortality rate and the functional outcomes assessed as by clinical remission defined as an Eckardt score ≤ 3 or if each item of the Eckardt score has improved by *>* 2 points.

**Table 1:** Eckardt score [9]

Score	Weight loss	Dysphagia	Chest pain	Regurgitation
0	None	None	None	None
1	< 5Kg	< 1/day	< 1/day	< 1/day
2	5 à 10 Kg	Daily	Daily	Daily
3	> 10 g	At each meal	At each meal	At each meal

**Table 2:** Modified Eckardt Score for Pediatrics [9]

Score	Weight	Dysphagia	Retrosternal pain (child > 2 years old)	Regurgitation Vomiting
0	Normal growth	None	None	None
1	Loss of 0,5 à 1 SD	Occasional	Occasional	Occasional
2	Loss between 1 et 2 SD	1 fois/jour		1fois/jour
3	Weight loss > 2 SD	Multi-daily		Multi-daily

The disease is considered active if the score is greater than 3. Clinical remission is defined by a score less than or equal to 3 or if each item has improved by more than 2 points.

**Table 3:** The Clavien-Dindo classification

Classification	Definition	Examples
Grade I	- Any deviation from the normal postoperative course without the need for pharmacological treatment or surgical, endoscopic and radiological interventions - Allowed therapeutic regimens are: drugs as antiemetics, antipyretics, analgetics, diuretics and electrolytes and physiotherapy. This grade also includes wound infections opened at the bedside.	Post operative ileus Hypokalemia Hypovolemia Vomiting Gastroparesis Diarrhea Parietal collection (seroma, hematoma, lipolysis)
Grade II	- Requiring pharmacological treatment with drugs other than such allowed for grade I complications - Blood transfusions and total parenteral nutrition are also included.	Surgical wound infection Pneumonia Urinary tract infections Deep vein thrombosis Severe anemia requiring transfusion Constipation Occlusion on flanges
Grade IIIa	Complication requiring surgical, endoscopic or radiological treatment without general anesthesia	Evisceration parietal suppuration Deep collection
Grade IIIb	Complication requiring surgical, endoscopic or radiological treatment under general anesthesia	Postoperative peritonitis Bowel obstruction Deep collection Fistula
Grade IV IVa IVb	Life-threatening complication (including CNS complications)* requiring IC/ICU-management	Visceral failure Pulmonary embolism Septic shock Postoperative peritonitis
	single organ dysfunction (including dialysis)	
	Multiorgan dysfunction	
Grade V	Death of a patient	

*brain hemorrhage, ischemic stroke, subarrachnoidal bleeding,but excluding transient ischemic attacks (TIA);IC: Intermediate care; ICU: Intensive care unit

### Statistical analysis

The study variables were entered into EPI info version 7. Quantitative variables were expressed as means and standard deviations, qualitative variables as percentage. The Chi-2 test was used to compare the groups and the proportions. The Student’s t-test was used for the analysis of variances for the comparison of the means. The threshold of statistical significance was set at a *p*-value less than 0.05.

## Results

During the 10-year retrospective study period, 31 cases of AC were managed surgically by Heller’s cardiomyotomy, yielding a frequency of 3.1 cases per annum. Of these 31 operative cases, two patients were excluded due to incomplete medical records and unavailable phone contacts. Hence, 29 patients were enrolled and constituted the study population.

### Epidemiological data

**Fig. 3:**
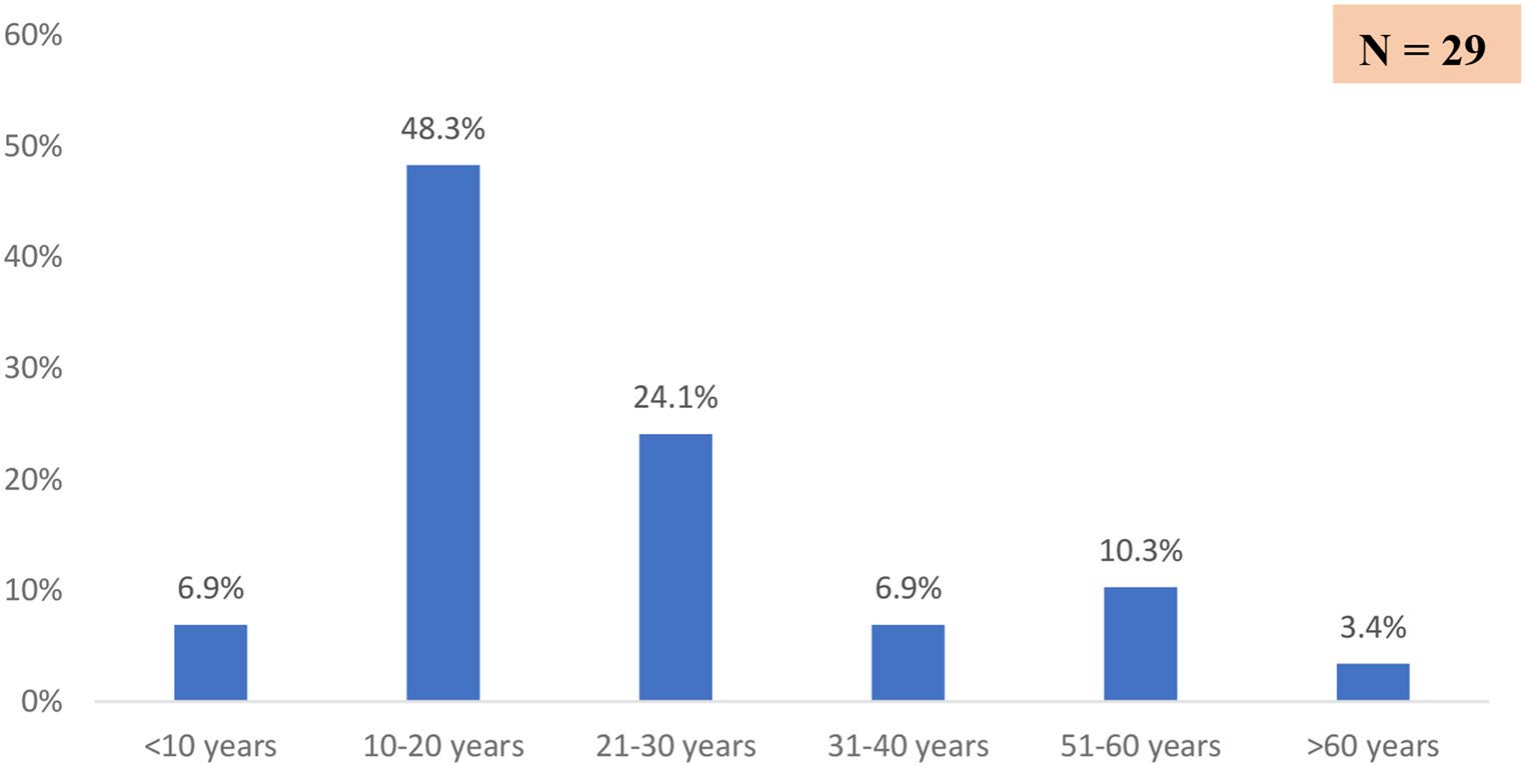
Distribution of cases by age

The mean of patients’ ages at symptoms onset was 24 ± 16 years (range: 8 months − 61 years). Patients aged 10 to 20 years were the most affected (48%) see Fig. 3. The age group under 10 years included two infants aged 08 and 12 months. Females were 3.8 times more affected than males; 79% vs. 21%.

### Preoperative data

Familial AC associated with congenital scoliosis was observed in one patient and Gougerot-Sjögren syndrome in another. Dysphagia was the first complaint at the time of symptom onset in 55% of patients followed by regurgitation in 21%. Jet-like vomiting was the initial symptom found in the two infants of our study population. Achalasia started during the first trimester of pregnancy by chronic food vomiting in a patient (see Table 4). At hospital presentation and before surgery, dysphagia, regurgitation and weight loss were present in all our patients. Dysphagia involved solids and liquids in 12 (44%) patients. The paradoxical type of dysphagia (dysphagia to liquids and not to solids) was not found. In 24 patients, the mean weight loss before HCM was 28 ± 5%. The two infants had a weight loss greater than 3 standard deviations (SD), suggestive of severe chronic malnutrition. The mean adult preoperative Eckardt Score was 9 ± 1.1 (range 7–11). Symptoms duration was 51 ± 20 months (range: 04–96 months). Overall, 59%, 38% and 3% of patients had symptoms duration of 5–9 years, 1–4 years and less than a year respectively.

**Table 4:** Distribution according to the inaugural symptom in our cohort

Symptoms	Numbers (%)
Dysphagia	16 (55.2)
Regurgitation	06 (20.7)
Chest pain	04 (13.8)
Projectile vomiting	02 (6.9)
Vomiting in pregnancy	01 (3.4)
Total	29 (100)

**Fig. 4:**
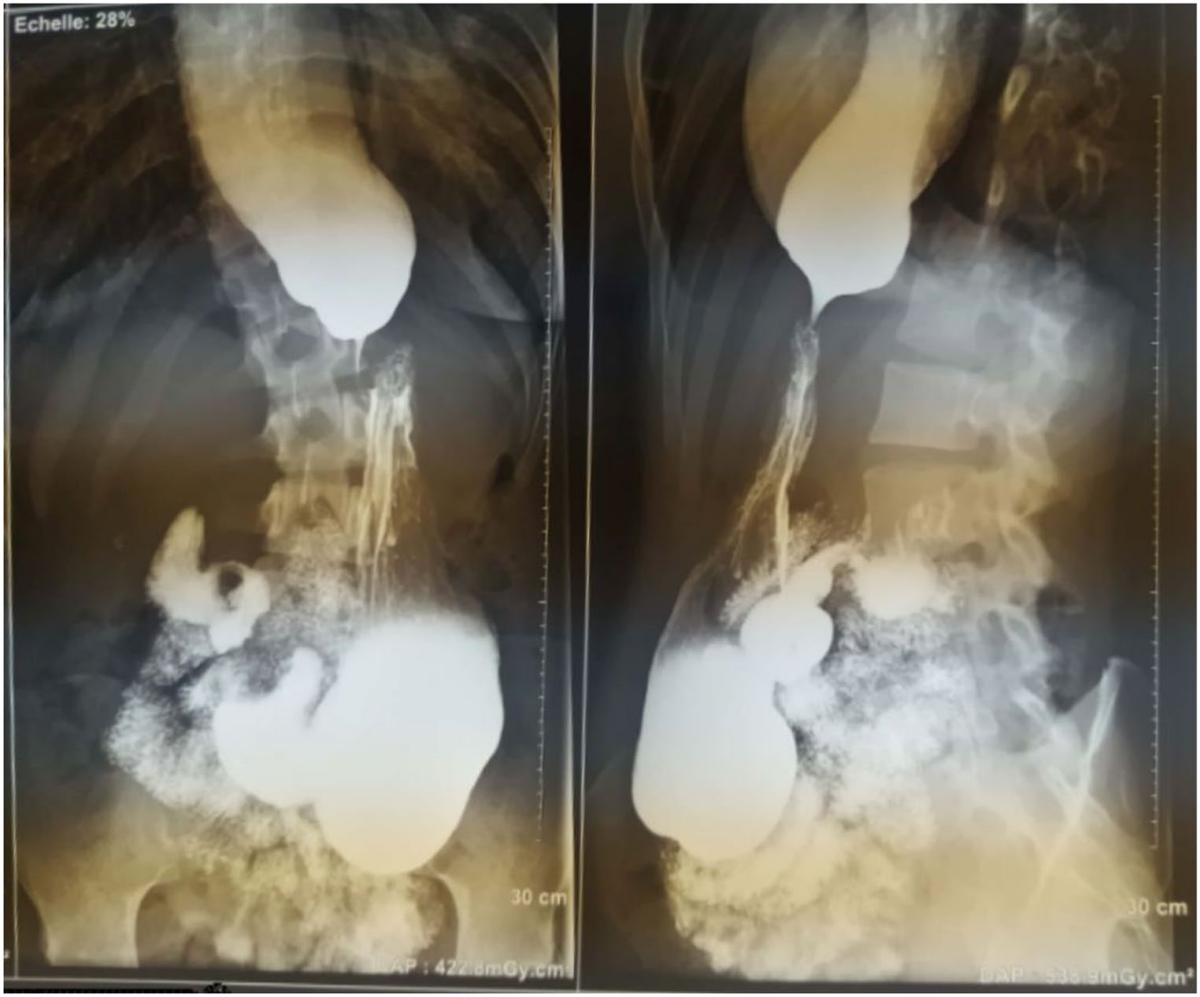
The barium swallow imaging of a patient before surgery showing esophageal dilation with barium retention, “bird’s beak” narrowing of the cardia and “snowflake-falling” filling of the stomach

Preoperatively, upper GI endoscopy was performed on 25 (86%) patients, of whom seven (28%) patients had no endoscopic signs of AC and were diagnosed using a conventional x-ray contrast imaging or “barium swallow test”. The endoscopic sensation of a tension area across the gastroesophageal junction, dilation and stasis were found in 64%, 60% and 56% of patients respectively. Only five biopsy and histology results were found and did not show a malignant process. Barium swallow was performed in 27 (93%) patients and showed non relaxation of the LES, stasis and dilation in all 27 patients. Among these 27 patients, esophageal dilation was quantified in 16 patients, which allowed us to obtain the radiological classification of AC according to Ressano Malenchini [9]. Esophageal dilation was between 4 and 6 cm (stage 2) and greater than 6 cm (stage 3) for 6 and 8 patients respectively. A tortuous esophagus (stage 4) was found in 2 patients. The median dilation was 07 cm with extremes ranging from 04 to 13 cm. Figure 4 shows esophageal dilation with barium retention, “bird’s beak” narrowing of the cardia and “snowflake-falling” filling of the stomach on a barium swallow before surgery. Megaesophagus was diagnosed in one patient during a contrast-enhanced thoracoabdominal CT-scan. The two aforementioned infants with AC were diagnosed using a barium swallow test. No patient had a diagnostic esophageal manometry done.

### Intraoperative data

Dilation was performed as first-line treatment in five (17%) patients. The latter conservative management immediately failed in three, while recurrence of AC post-pneumatic dilation at 06 months and 20 months was seen in two, thus, warranting surgical treatment by HCM. Overall, first-line treatment by surgery was performed in 83% of patients. ASA II and III involved 25 and 04 patients respectively. Laparotomy was the most used approach in 76% of cases versus 24% of laparoscopies. The median of the esophagocardiomyotomy length was 10 cm. The lengths on the esophagus, the cardia and on the stomach have not been specified. The most prevalent antireflux systems after myotomy were Dor’s anterior, Toupet and Nissen fundoplications in 17, 09 and 03 patients respectively. Perforation of the esophageal mucosa was the only intraoperative complication in eight (28%) patients; two during laparoscopy and six during laparotomy. All perforations were repaired with simple sutures.

### Post-operative data

A repeat conventional x-ray contrast imaging was done in six patients. The indication was to verify the absence of esophageal leakage before the removal of the nasogastric tube in four patients who had intraoperative mucosal perforation. In the remaining two cases it was for routine postoperative evaluation. These esophagograms revealed an absence of esophageal fistula, normal-sized cardias and homogeneous filling of the stomach. Nasogastric tubes were removed average at 3 ± 2 days (range 1–10 days). Gradual oral feeding was started immediately after nasogastric tube removal. The time to the disappearance of symptoms was immediate (as soon as oral feeding was resumed) in 21 patients and four patients presented intermittent dysphagia during the first two postoperative weeks. The average length of hospital stay was 6 ± 2 days, with no statistically significant difference depending on the surgical approach; 6 ± 2 and 5 ± 2 respectively for laparotomy and laparoscopy. All patients were followed up for a postoperative period ranging from 03 to 12 months with a minimum follow-up of 06 months. Three (10%) patients presented with postoperative complications. These were patients who had cardiomyotomy by laparotomy. These were two superficial wound infection and one wound evisceration rated respectively as grade I and grade III Clavien-Dindo postoperative complications. Only the case of post-operative evisceration required re-laparotomy with excellent postop recovery. No death was recorded at three months of follow-up. After a one-year postoperative follow-up, only one out of 26 patients presented with occasional burning chest pain, yielding a short-term remission rate of 96% (Table 5). Generally, all patients had a gain in weight after surgery. The medians in the percentage of weight gain at one, three, six- and 12-months post operation were respectively 12%, 25%, 33% and 36%. Lastly, the short-term clinical remission rate according to the Eckardt score was 100%, average score of 1.5 ± 0.5 (vs. preoperative Eckardt Score of 9 ± 1; *p* < 0.0001).

**Table 5:** Clinical remission at 6 months and 1 year after surgery/HCM

Short-term follow-up		Symptoms		
		Dysphagia	Regurgitation	Chest pain
6 months	Preoperative (*n* = 29)	29	29	29
	Postoperative (*n* = 29)	-	1	2
	Rate (%)	100	96,6	93,1
12 months	Preoperative (*n* = 29)	26	26	26
	Postoperative (*n* = 29)	-	-	1
	Rate (%)	100	100	96,2

## Discussion

In this retrospective cohort study, we aimed to report recent findings on the perioperative outcomes of Heller’s cardiomyotomy for AC from 29 African patients in Cameroon.

We observed that females were 3.8 times more affected. A similar female predominance has been reported in Cameroon and Madagascar [6, 16]. The average age of onset of symptoms was 24 years. This age is almost identical to that found in a series of 251 women [18].

We found long symptom duration for AC with suboptimal but acceptable diagnostic tests. Laparotomy was the most used surgical approach for HCM. Mucosal perforation was the main intraoperative complication and Dor’s anterior partial fundoplication was the most common antireflux procedure. Postoperative morbidity was slight and mortality was zero. Postoperative complications were found only in patients operated by laparotomy. Clinical remission after a one-year postoperative follow-up was almost complete.

Similar to several other authors [16, 19, 20] we observed late AC diagnoses which could be explained by the intermittent and fluctuating nature of the dysphagia, the tolerance and adaptation of the patients to the symptoms, but also because of a normal endoscopy. The relative rarity of AC could also explain long symptom duration because physicians often have a low index of clinical suspicion. In this study setting, symptom duration was probably made longer due to the unavailability of esophageal manometry, the reference diagnostic test for AC [21, 22]. Findings of upper GI endoscopy performed before and/or during HCM showed a normal esophageal mucosa. This corroborates previous reports stipulating that upper GI endoscopy is a low-sensitivity test that misses out on 40% of ACs [9]. Nevertheless, upper GI endoscopy is crucial in the diagnostic process, because it helps rule in or rule out any associated malignant transformation or pathology. Conventional x-ray contrast imaging has a more diagnostic sensitivity for AC compared to upper GI endoscopy and the combination of both of these tests may yield promising better accurate diagnostic performances for resource-constraints environments.

HCM was performed via open surgery trice more than laparoscopy, explained by the growing embryonic practice of the latter in Cameroon. Dor’s anterior partial fundoplication was the most performed antireflux procedure because it was performed in all patients with an intraoperative perforation. Also, the obsession of surgeons in preventing esophageal fistula is another explanation for the preponderance of Dor’s anterior partial fundoplication. Unlike laparoscopic HCM, open HCM had postoperative complications and a longer length of hospital stay. As such, the benefits of laparoscopic HCM should suggest this approach as a first step [14].

We acknowledge a few study limitations like the retrospective study design which hindered a complete appraisal of the scope of AC because we could not precisely report the total number of patients with AC who presented to our study setting, as well as the total number operated and not operated who received other treatments. We reported only cases of AC from the surgical units (31 patients). Likewise, the retrospective nature of the current study led us to exclude two patients with AC due to incomplete records. However, we retained a relatively high response rate of 93%. Furthermore, the retrospective study design hindered us from studying the histology of the esophageal mucosa since findings of esophageal biopsies were not reported in the medical records of patients. The statistics were used to suggest potential differences to study in the futures with an adequately powered study. Nonetheless, based on well followed-up patients, we used a cohort design to provide a recent and contributive level of scientific evidence to the scarcity of data on the perioperative outcomes of HCM for achalasia in the African region. These findings may guide surgeons making informed decisions in their therapeutic strategies for AC in resource-constraint settings.

## Conclusion

The frequency of AC is increasing in Cameroon and affects the female sex more often for as yet unknown reasons. AC is often diagnosed quite late in this low-resource setting. The realization of the upper GI endoscopy -conventional x-ray contrast imaging couple when faced with clinical suspicion of AC could reduce this symptoms duration geared at improving therapeutic outcomes. The surgical treatment of AC by HCM yields satisfactory outcomes, especially via laparoscopic management. An improvement in diagnostic esophageal manometry and mini-invasive surgical infrastructure and the required surgical training/skills are needed for optimal AC care. High-quality large multi-center studies comparing laparoscopic to laparotomy HCM for treating AC in Africa are warranted.

## Data Availability

The datasets used and/or analyzed during the current study are available from the corresponding author on reasonable request.

## Abbreviations

AC: Achalasia of the cardia
LES: Lower esophageal sphincter
HCM: Heller’s cardiomyotomy
GI: Gastrointestinal
EBS: Endoscopic Biliary Sphincterotomy
